# Fluid prescription practices of anesthesiologists managing patients undergoing elective colonoscopy: an observational study

**DOI:** 10.1186/1756-0500-7-356

**Published:** 2014-06-10

**Authors:** Laurence Weinberg, Matthew Faulkner, Chong O Tan, Daniel H Liu, Stanley Tay, Mehrdad Nikfarjam, Philip Peyton, David Story

**Affiliations:** 1 Anesthesiologist, Department of Anesthesiology, Austin Hospital, Melbourne, Victoria, Australia; 2 Registrar, Department of Anesthesiology, Austin Hospital, Melbourne, Victoria, Australia; 3 Surgeon, Austin Hospital, Melbourne, Victoria, Australia; 4 University of Melbourne, Melbourne, Victoria, Australia

**Keywords:** Colonoscopy, Fluid intervention, Pharmaco-economics, Complications, Anesthesia

## Abstract

**Background:**

Routine fluid prescription is common practice amongst anesthesiologists caring for patients undergoing colonoscopy. However there is limited information about routine procedural fluid prescription practices of anesthesiologists in this setting. Routine fluid administration may also have important pharmaco-economic implications for the health care budget. Therefore we performed a prospective observational study assessing the fluid prescription practices of anesthesiologists caring for patients undergoing elective colonoscopy.

**Methods:**

With Institutional Review Board approval, adult patients receiving procedural fluid intervention during elective colonoscopy were included. Data collected: size of intravenous cannula inserted, volumes of fluid administered, adverse events, procedure duration, and pharmaco-economic costs associated with fluid prescription. Anesthesiologists and gastroenterologists were blinded to the study.

**Results:**

We collected data on 289 patients who received fluid prescription by their attending anesthesiologist. Median patient age: 48 yrs (range 18–83), gender: 174 (60%) female; median duration of procedure: 24 minutes (range 12–48). Cannula size: 181 (63%) patients received a 22G cannula or smaller. Median volume of fluid administered during the colonoscopy was 325 ml (range 0 to 1000 ml). Median duration of the procedure: 25 minutes (range 12 to 48 minutes). Median volume of fluid administered in the post anaesthesia recovery unit: 450 ml (range 0 to 1000 ml). Fifteen patients (5%) became hypotensive during the procedure and two patients (<1%) developed hypotension in the PACU. There was no difference in the median fluid requirements between patients with hypotension and those without. Fluid volumes were strongly associated with increasing cannula diameter (p = 0.0001), however there was no association between fluid volumes administered and vasopressor use, peri-procedural adverse events, or procedure duration. At our institution fluid therapy currently cost about AUD$4.90 per patient: 1 L crystalloid $1.18 and fluid delivery set $3.77 Our institution performs over 9000 endoscopic procedures annually with fluid therapy costing about $45,000/year.

**Conclusions:**

Routine fluid prescription by anesthesiologists managing patients undergoing colonoscopy was ineffective with low actual fluid volumes delivered during the procedure. There was no association between volumes of fluid delivered and procedural hypotension, adverse events, or procedure duration. Anesthesiologists should question the clinical and pharmaco-economic value of routine fluid administration for patients undergoing elective endoscopy.

## Background

Colon cancer screening programs worldwide are adding to greatly increased demand for colonoscopy, making it one of the most common procedures performed [1-3]. Intravenous (IV) fluid administration for patients undergoing colonoscopy is common practice amongst anesthesiologists despite the lack of evidence for reduction of intra-procedural hypotension or post-procedural morbidity outcomes [4]. Fluid administration in this setting allows a continuous flush line for the administration of anesthesia drugs, and may facilitate faster recovery and promote patient satisfaction. Clinically measureable adverse outcomes of fasting and bowel preparation (such as hypotension, drowsiness, nausea, vomiting, dehydration, dizziness and thirst) have been widely documented [5-8], but there is limited data from randomized studies examining routine fluid management practices during colonoscopy, and the role of fluid prescription in the prevention of such events continues to be poorly understood [9,10]. There is additional cost and workload if fluid is to be routinely administered to all patients [11], and emerging evidence that fluid administration to patients undergoing colonoscopy may not reduce peri-procedural adverse events [4]. Routine fluid administration may have significant pharmaco-economic implications taking into consideration the cost of the fluid flasks and giving sets. Therefore we performed a prospective observational study assessing the routine fluid prescription practices of anesthesiologists caring for patients undergoing elective colonoscopy.

## Methods

With approval from the Human Research Ethics Committee at Austin Health (H2013/05078), we conducted a prospective, blinded observational study of patients undergoing elective colonoscopy at the Austin Hospital. Austin Hospital is a university hospital in Australia with tertiary level services in cardiothoracics, major hepatobiliary surgery including liver transplantation and spinal injury. In addition to all major surgical specialties, the hospital provides services for over 9,000 endoscopic procedures annually. All adult patients undergoing elective colonoscopy who received peri-procedural IV fluids as part of standard anesthesiology care were included. Patients undergoing combined gastroscopy and colonoscopy, emergency colonoscopies, and colonoscopies performed without IV fluid being prescribed were excluded. In accordance with the requirements of the NHMRC National Statement on Ethical Conduct in Human Research 2007, Austin Health's Research Ethics Committee waived the requirement for patient consent.

We collected data on timing fluid administration (i.e. prior to arrival in the operating room, in the operating room but before procedure commencement, or post procedure commencement), the size of the peripheral cannula through which fluid was delivered, and volumes of fluid administered both during the procedure and in the post anesthesia recovery unit (PACU). Other data collected included procedure duration, incidence of hypotension, nausea, vomiting, and the use of vasopressors to treat or prevent hypotension. Hypotension was defined as any documented systolic blood pressure less than 20% of the pre-procedural value. Nausea was defined as any patient reporting a feeling of sickness in the stomach with an urge to vomit, or the use of any antiemetic drug during the colonoscopy procedure admission. If a patient received an antiemetic drug they were considered to have experienced nausea during the admission. Vomiting was defined as the ejection of any contents of the stomach through the mouth at any point once the procedure was completed. Finally pharmaco-economic costs associated with peri-procedural fluid intervention were calculated, taking into consideration the number of IV giving sets prepared by the nursing staff per list and the total amount of time taken to prepare these fluids. All procedural anesthesiologists and gastroenterologists, as well as the recovery nurses were blinded to the study.

As per our institution’s protocol for elective colonoscopy, all patients received a standardized bowel preparation regime that included a modified diet 3 days before the procedure, then 3 sachets of PicoPrep (Sodium picosulfate, Fresenius Kabi, NSW) the day before the procedure with 250–500 ml water, then free clear fluids up until four hours before the procedure. Small sips of water on the morning of surgery were allowed for all medications, except for oral hypoglycemics, which were stopped the night before. All patients were admitted to hospital on the day of the procedure. Trained anesthesia nurses were responsible for the preparation of the IV fluid and giving sets. As per our institution’s protocol for elective colonoscopy, all patients require a 30 minute recovery period in PACU before being discharged back to the departure lounge for a light snack and drink before being discharged home.

All patients had an IV cannula (Introcan Safety cannula, B. Braun Medical Industries Sdn, Penang, Maylasia) inserted by the attending anesthesiologist. The size of the IV cannula was at the discretion of each attending anesthesiologist. As per hospital protocol, the type of fluid used was a 1000 ml balanced crystalloid solution flask, (Hartmann’s Solution, Baxter Healthcare, Toongabie, NSW), but the amount of fluid delivered was again entirely at the discretion of the attending anesthesiologist. Patients were placed in a left lateral position with their knees flexed. Consultant anesthesiologists performed the procedural sedation and consultant gastroenterologist performed all the endoscopy procedures. As per hospital protocol, routine monitoring consisted of 3 lead electrocardiography, oxygen saturation, and non-invasive blood pressure measurements taken from the left upper arm every 3 minutes. Oxygen was delivered via facemask at 6 L/min with the option of attaching capnography to these masks if clinically indicated. All patients underwent a standardised conscious sedation technique using propofol IV (Fresenius Kabi Australia, Pty Ltd, NSW) (1 mg/kg bolus, followed by 30–50 mg boluses) and fentanyl IV (Aspen Pharmacare, Australia Pty Ltd, NSW) (0.5 to 1 ug/kg boluses) titrated to effect. As per hospital guidelines, the management of peri-procedural hypotension consisted of boluses of metaraminol IV 0.5 mg (Sandos, NSW) or ephedrine 5 to 10 mg IV (Hospira, Victoria). The use of all other emergency drugs including atropine or adrenalin was permitted if clinically indicated. After the procedure patients were transferred to the PACU for a 15-minute observation period, and if clinically stable were discharged back to the day admission unit for a light diet and free fluids before being discharged home. The treatment of hypotension in recovery was at the discretion of the attending anesthesiologist and consisted of further fluid boluses, or with the same pharmacological intervention described above. All data collected were transcribed onto a data collection form and then entered into a de-identified electronic database.

### Statistical analyses

A statistical software package (SPSS Version 19.0; IBM Co, Armonk, NY, USA) was used for statistical analysis. Results were expressed as either a median (range), or in the form of frequencies and proportions unless otherwise stated. Multivariate analysis was undertaken using a backward stepwise logistic regression model to identify factors independently associated with fluid intervention including all factors where the p-value was less than 0.1 on univariate analysis. The 95% confidence intervals (CI) were reported where appropriate. A p-value < 0.05 was considered significant. We reported this study using the STROBE guidelines for reporting observational studies [12].

## Results

**Figure 1:**
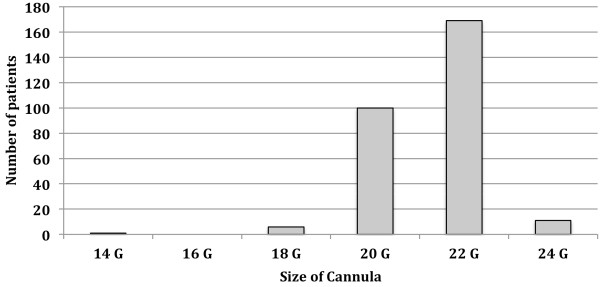
Cannula sizes inserted by the attending anesthesiologist for peri-procedural IV fluid delivery.

**Figure 2:**
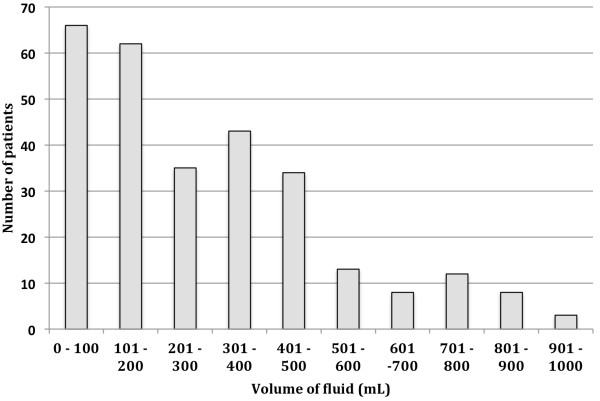
The average amount of fluid delivered during elective colonoscopy.

We collected detailed fluid prescription practices of anesthesiologists for 289 patients undergoing elective day case colonoscopy procedure over a 3-month period. Median patient age was 48 years (range 18–83 years); 174 (60%) were female. One-hundred and eighty one patients (63%) received a cannula size 22 gauge (internal diameter 0.41 mm) or smaller; and 108 patients (37%) received a 20 gauge cannula (internal diameter 0.60 mm) or larger (Figure 1). All patients had their IV cannulae inserted in the procedure room before the colonoscopy procedure commenced. The median volume of fluid administered from time arrival in the operating room to completion of the colonoscopy was 325 ml: range 0 to 1000 ml (Figure 2). The median duration of the procedure was 25 minutes (range 12 to 48 minutes). The median volume of fluid administered in the PACU was 450 ml: range 0 to 1000 ml.

Fifteen patients (5%) became hypotensive during the procedure and two patients (<1%) developed hypotension in the PACU. There was no difference in the median fluid requirements between patients with hypotension and those without hypotension (290 ml vs. 327 ml, p = 0.52). Three patients (1%) experienced nausea in the PACU. All patients responded to simple pharmacological intervention. Patient groupings for statistical comparison were created about the median volume of fluid administered. The associations of patient demographics, intra-procedural data and fluid administration were evaluated using both univariate and multivariate analyses (Tables 1 & 2). The only independent predictor of higher volume fluid administration was the cannula size (p < 0.001). There was no association between higher volumes of fluid administered and patient age (p = 1.00) and gender (p = 0.33), duration of procedure (p = 1.00) or peri-procedural hypotension (p = 1.00). On average four IV fluid giving sets were prepared per list, taking on average 4.78 minutes for preparation. This represents approximately 1.3 min spent per flask of IV fluid preparation. At our institution fluid therapy currently cost, in Australian Dollars (0.95 US Dollars) about $4.90 per patient: 1 L crystalloid - $1.18 and the fluid delivery set - $3.77.

**Table 1:** Univariate regression analysis of patient and procedural data and their relationship to the median volume of fluid administered

**Variable**	**Lower fluid volume (<325 ml) n (%)**	**Higher fluid volume (>325 ml) n (%)**	**p-value**
**Total (n = 289)**	166 (57)	123 (42.5)	-
**Age**	-	-	1.000
<50 yrs (n = 159)	91 (57)	68 (43)	
>50 yrs (n = 130)	75 (58)	55 (42)	
**Gender**	-	-	0.330
Male (n = 115)	70 (62)	44 (38)	
Female (n = 174)	96 (55)	78 (45)	
**Hypotension**	-	-	1.000
Yes (n = 17)	10 (59)	7 (41)	
No (n = 272)	156 (57)	116 (43)	
**Cannula size**	-	-	0.0001*
≤22 g (n = 181)	120 (66)	61 (34)	
≥20 g (n = 108)	47 (44)	61 (56)	
**Duration**	-	-	1.000
<25mins (n = 105)	61 (58)	44 (42)	
>25mins (n = 61)	35 (57)	26 (43)	

**Table 2:** Multivariate logistical regression analysis of patient and procedural data and their relationship to fluid volumes administered

	**Odds ratio (95% CI)**	**p-value**
Age: >50 yrs	0.95 (0.48 to 1.87)	0.881
Gender: Female	2.01 (0.98 to 4.13)	0.057
Hypotension: yes	1.98 (0.52 to 7.52)	0.315
Duration	1.15 (0.58 to 2.28)	0.680
Cannula size: ≥20 gauge	2.70 (1.64 to 4.45)	<0.001*

## Discussion

We performed a prospective observational study assessing fluid prescription practices amongst consultant anesthesiologists caring for patients undergoing elective day surgery colonoscopy. Intravenous fluid administration was not associated with less hypotension, and on average only 325 ml of IV fluid was administered during each procedure. When allowing for the large number of colonoscopies performed each year (more than 500,000 in Australia and more than 10 million in the United States), routine use of intravenous fluid prescription for elective colonoscopy has considerable pharmaco-economic implications.

The findings of our study are comparable to the finding of Leslie et al. [4]. In their study, the “low-volume” fluid intervention group received 1.5 ml/kg Hartmann’s solution pre-operatively and “high-volume” fluid intervention group received 15 ml/kg Hartmann’s solution. Similar to our study were no differences between high and low volume fluid intervention groups in the prevention of hypotension. Similarly, Yogendran et al. [6] showed that, for day stay patients, high volumes of preoperative intravenous fluid administration (20 ml/kg) reduced the incidence of dizziness, drowsiness and thirst but there was no difference in the hemodynamic parameters or the discharge times. The rate of hypotension in our study was 5%, which is higher than the quoted 0.1% incidence of hypotension reported by Waye et al. [13] These investigators prospectively evaluated the complications of patients undergoing colonoscopy. However, the definition of hypotension in that study was poorly defined. In contrast, in a similar study Lancaster et al. [14] evaluated patients undergoing lower endoscopic procedures and reported a 17% incidence in severe hypotension, defined as a greater than 40% fall in systolic blood pressure from baseline. The dose of sedative/anesthesia drugs, as well as patient age did not correlate with hypotension. However, in their study the duration of the procedure was strongly correlated with the incidence of hypotension, a finding that was not observed in our study. Unfortunately, Lancaster et al. did not report amount of fluid prescribed [14].

As expected, the volumes of fluid delivered to patients in our study correlated strongly with the IV cannula size, where patients with a larger cannula received a larger volume of fluid. However the volumes of fluid delivered in this study fall well short of the maximum possible volumes delivered through the cannulas (Table 3). The average duration of colonoscopy during this study was 25 minutes and on average only 325 ml of fluid was given per case. Further, 85% patients received less than 500 ml of fluid throughout the procedural period. The flow rate through any given cannula is dependent on pressure gradient between the IV fluid and the patient’s vein and also the resistance that develops within the IV fluid set, the IV cannula and the patient’s vein. Other factors that can reduce flow rates include kinks within the line, air or precipitates in the cannula, patient limb positioning, quality and size of the patient’s vasculature, viscosity of the fluid (crystalloid vs. colloid), and height of the fluid above the vein. The small volume of fluids that were administered during each procedure in our study is unlikely to have had any clinically beneficial effect on the patients’ haemodynamic status.

**Table 3:** Approximate maximum flow rates through different size cannulas

**Cannula size**	**Maximum Flow rate (mls/min)**	**Maximal volume of fluid that can be delivered for a given 25 min procedure**
24 gauge	22	550 mls
22 gauge	35	875 mls
20 gauge	60	1500 mls
18 gauge	105	2625 mls
16 gauge	215	5375 mls
14 gauge	345	8625 mls

At our institution, the cost of IV fluid therapy, in Australian Dollars (0.95 US Dollars, October 2013) is approximately $4.90 per patient: 1 L crystalloid $1.18 and fluid delivery set $3.77. Our institution performs over 9,000 endoscopic procedures annually and if every patient received IV fluid therapy it would cost the hospital approximately $45,000/year. In addition, on average 4 fluids sets are prepared by nursing staff with an average time taken per list to be 4.8minutes. We have approximately 20 endoscopy lists per week resulting in roughly 95minutes of nursing time spent on preparing fluids every week. For 1year this would roughly equate to 82 nurse hours. As IV fluids are typically prepared at the start of each list, the additional 5minutes saved from not preparing fluids may translate into earlier start times and increased patient turnover.

There are several limitations to our study. Although this is one of the largest prospective studies to report of detailed standard of care fluid intervention practices of anesthesiologists, only 289 patients were reviewed. However, this is the first study to report on routine peri-operative fluid prescription practices in a tertiary care hospital, reflecting actual anesthesia practices in this setting. It was possible that not all complications were properly recorded, or there were inaccuracies in the recording of fluid prescription data. However, all patient records were checked by two independent study investigators, and we consider the influence of inaccuracy to be insignificant. In addition, two investigators reviewed all anesthesia and PACU charts to ensure a thorough and accurate tally of fluid prescription and complications were documented. This is a single-centre study, which limits the external validity of our findings. However, our hospital shares similar characteristics with other tertiary hospitals in Australia. We did not calculate the cost of treating hypotension with the use of the pharmacological agents described above. Routine use of ephedrine or metaraminol for the treatment of hypotension also has pharmaco-economic considerations. At present, a single 10 mg/ml metaraminol ampoule (Sandos®, NSW) costs our institution Au$28.39, and the cost of a single 30 mg/ml ephedrine ampoule (Hospira®, Victoria) is Au$15.38. Similarly we have not recorded the pharmaco-economic implications of post-operative nausea and vomiting, or evaluated the effects of these adverse events on discharge times. This may also have direct pharmaco-economic implications, however the primary objective of this study was to document real-life and accurate fluid intervention practices, and determine if these would influence haemodynamic parameters such as hypotension. Finally, we have not made any economic assessment of stay in the PACU or day procedure unit. However, these circumstances in no way affect the validity of the results or the objectives of this study.

There are various strengths in this study. The quality of treatment that the patients receive is in keeping with the current best standard of care with specialist anesthesiologists, gastroenterologists and nursing staff who are adequately trained in caring for patients undergoing routine colonoscopy in this setting. Patient management in our endoscopy suite and PACU follows the Australia and New Zealand College of Anesthetists guidelines for management and monitoring of sedated patients and post anesthetic care [15,16], which is consistent with most hospitals in Australia and New Zealand. The advantage of conducting a study with clinicians being unaware of fluid data being collected reduces the risk of the Hawthorn effect, therefore the management of these patients reflects standard care. We have included all adult elective patients over a 3-month period, which would make the results of the study more consistent with the daily clinical practice.

## Conclusions

In conclusion, we observed that despite routine IV fluids being prescribed for patients undergoing elective colonoscopy, on average only 325 ml was actually delivered during each procedure. As expected, fluid volumes were strongly associated with increasing cannula diameter, however there was no association between fluid volumes administered and vasopressor use, peri-procedural adverse events, or procedure duration. At our institution fluid therapy currently costs about au$4.90 per patient. We think anesthesiologists should question the clinical and pharmaco-economic value of routinely administering intravenous fluids to adult patients undergoing elective endoscopy.

## Competing interest

No external funding and no competing interests declared.

## Authors’ contributions

LW: Principle Investigator who designed the study as part of an “Austin by Design” initiative. He was responsible for the ethics submission, analysis of data, and writing of the manuscript. COT: responsible for acquisition of data, literature review and writing of manuscript. MF, DL & ST: participated in data collection, entry of data from the medical records, checking of electronic medical records, and collation of final database. PP & DS: analyses of data and writing of manuscript. All authors read and approved the final manuscript.
